# Transmission and Control of African Horse Sickness in The Netherlands: A Model Analysis

**DOI:** 10.1371/journal.pone.0023066

**Published:** 2011-08-05

**Authors:** Jantien A. Backer, Gonnie Nodelijk

**Affiliations:** Department of Epidemiology, Crisis Organisation and Diagnostics, Central Veterinary Institute of Wageningen UR, Lelystad, The Netherlands; University of Liverpool, United Kingdom

## Abstract

African horse sickness (AHS) is an equine viral disease that is spread by *Culicoides* spp. Since the closely related disease bluetongue established itself in The Netherlands in 2006, AHS is considered a potential threat for the Dutch horse population. A vector-host model that incorporates the current knowledge of the infection biology is used to explore the effect of different parameters on whether and how the disease will spread, and to assess the effect of control measures. The time of introduction is an important determinant whether and how the disease will spread, depending on temperature and vector season. Given an introduction in the most favourable and constant circumstances, our results identify the vector-to-host ratio as the most important factor, because of its high variability over the country. Furthermore, a higher temperature accelerates the epidemic, while a higher horse density increases the extent of the epidemic. Due to the short infectious period in horses, the obvious clinical signs and the presence of non-susceptible hosts, AHS is expected to invade and spread less easily than bluetongue. Moreover, detection is presumed to be earlier, which allows control measures to be targeted towards elimination of infection sources. We argue that recommended control measures are euthanasia of infected horses with severe clinical signs and vector control in infected herds, protecting horses from midge bites in neighbouring herds, and (prioritized) vaccination of herds farther away, provided that transport regulations are strictly applied. The largest lack of knowledge is the competence and host preference of the different *Culicoides* species present in temperate regions.

## Introduction

African horse sickness (AHS) is a vector-borne viral disease that can affect all species of equines. In zebras and donkeys the clinical symptoms are often mild [1], as is seen in endemic areas in sub-Saharan Africa. But when the virus is introduced in naive horse populations the morbidity and mortality rates may exceed 90% [1]. Most notably the 1987–1991 epidemic on the Iberian peninsula and Morocco caused the death of 2000 horses and required a considerable vaccination effort to eradicate the disease [2].

The AHS virus is closely related to the bluetongue virus (Reoviridae: *Orbivirus*) and is transmitted by the same vector genus (midges or *Culicoides*). For a long time it was thought that bluetongue could not be transmitted by vector species in more temperate regions of Europe (above 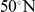), until a bluetongue epidemic of serotype 8 occurred in this region in 2006 [3], [4]. In the Iberian epidemic (1987–1991) AHS virus was isolated from pooled samples of vectors that contained *C. obsoletus* and *C. pulicaris*, species that both occur in northern Europe [5]. This suggests that AHS could be spread by a competent vector in this region, forming a serious threat for local horse populations. With an estimated number of 450 000 horses [6], The Netherlands is one of the most densely horse-populated countries in Europe (on average 11 horses/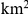). An AHS epidemic could have a devastating effect on this population, leading to large economic losses and substantial social impact. For this reason it is important to comprehend the speed and extent of AHS virus transmission, as well as the effect of control measures, to be optimally prepared for an AHS virus introduction.

**Figure 1:**
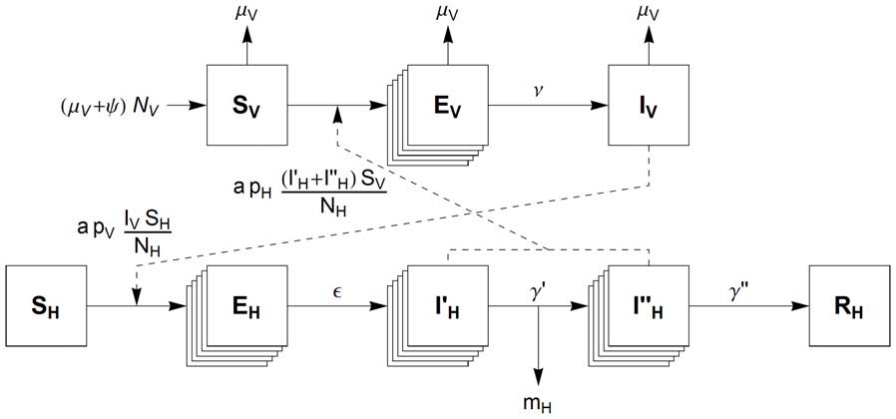
Schematic representation of the vector-host model. Animals are either in a susceptible (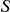), latently infected (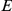), infectious (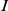) or recovered (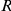) state, with subscripts 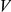 or 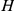 indicating the vector or host. The stacked squares denote that this compartment is divided into multiple stages. Here 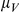 is the vector mortality rate, 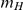 the host mortality due to the disease, 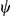 the adaptation rate of the vector population, 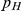 and 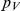 the transmission probabilities from host to vector and from vector to host, 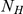 and 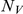 the total host and vector population size, 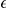 and 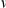 the rate of becoming infectious for hosts and vectors, and 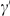 and 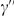 the leaving rate from the first and second infectious host classes.

As the AHS virus has never been found in The Netherlands, a model can help to study transmission and control of AHS. Model analysis can provide insight in the main parameters that determine whether and how the disease will spread. Lord et al. have largely contributed to the modelling of AHS [7]–[9], albeit with limited computational power and few experimental results. Since the bluetongue epidemic in 2006 more information has been collected on transmission of Orbiviruses [10] and on local midge densities that vary in time and space [11]. We adapt a basic vector-host model to match the infection biology (Figure 1) and use the new information for estimating the model parameters for the Dutch situation (Table 1). Next, we extend the model to estimate the virus transmission to other herds, by introducing a diffusion term for midges migrating to neighbouring herds. This theoretical diffusion model ignores the transmission route via transport of infected horses that are prohibited after the disease has first been detected. An observed epidemic could provide sufficient information to justify the use of stochastic simulations, as has been done for bluetongue in Great Britain [12]–[14]. But because of the lack of outbreak data and the large uncertainties for AHS, we restrict our approach to deterministic simulations.

**Table 1:** Parameters used in the AHS transmission model and their default values.

	parameter	symbol	default	5%–95%	distribution
			value	range	function
	latent period hosts (days)	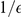	3.7	2.5–4.9	Normal
	no. of stages for latent host class	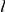	16		
	average infectious period dying hosts (days)^a^	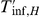	4.4	2.2–6.6	
	overall recovery rate from 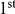 infectious host class ( 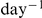 ), 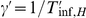	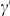	0.23	0.15–0.45	
	no. of stages for 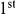 infectious host class	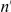	19		
*	average infectious period recovering hosts (days)^a^	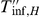	6.0	3.0–9.0	Normal
	overall recovery rate from 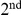 infectious host class ( 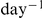 ), 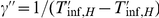	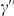	0.63	0.42–1.25	
	no. of stages for 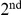 infectious host class	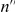	10		
	mortality hosts	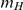	0.70	0.43–0.97	Uniform
	temperature in August (  C)	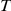	17.2	12.1–22.4	Normal
*	blood feeding interval (days), Eq. 3	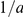	7.5	4.7–17.7	
	extrinsic incubation period (days), Eq. 3	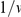	16	9.2–48	
	no. of stages for incubating vector class	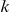	10		
*	average life span (days), Eq. 3	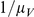	22	16–31	
	correction rate vector population	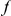	10		
*	transmission probability host to vector	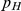	0.04	0.01–0.1	Gamma
*	transmission probability vector to host	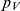	0.77	0.50–0.95	Beta
	vector to host ratio in August	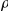	226	1–4219	Weibull
	host population size	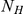	66	32–100	Normal
	distance between host groups	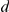	2.4	1.4–10.8	Eq. 4 in Text S1
	vector diffusion coefficient ( 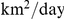 )	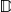	1.12	0.89–1.36	Normal

Ten key parameters are varied in the uncertainty and sensitivity analysis, according to their distribution functions.

* Five parameters are varied in the control analysis, their reduction ranging from 0 (no effect) to 1 (full effect).

a The length of the average infectious periods of dying and recovering hosts have a fixed ratio: 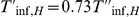.

Using our model, we aim to study how the absence or presence of epidemics is affected by the model parameters and the time of introduction and how the epidemic behaviour is affected by the model parameters. Furthermore, we aim to study how the epidemic behaviour is affected by reduced model parameters due to control measures. Taking the variation and uncertainty in the model parameters into account, gives the full range of expected outcomes and identifies gaps in the current knowledge of AHS transmission. The effect of modifying parameters that could to some extent be controlled during an epidemic is evaluated to assess the effectivity of control measures. Although no registered vaccine is available in The Netherlands at the moment, we will also take vaccination into account as the EU may allow its use for epidemic control purposes [15].

## Methods

### Vector-host model

The vector-host model is a deterministic compartmental model [16] for one host species and one vector species. It is similar to the basic model of Lord et al. [7], but differs on three points. First, a latent compartment is included for the hosts as experiments have shown that their latent period is not much shorter than their infectious period. Secondly, the length of the infectious period differs for dying and recovering hosts. And thirdly, some compartments are divided into multiple stages, in such a way that the gamma distributed residence time agrees with experimental data.

Figure 1 shows the different compartments and the relations between them schematically. A host can be either susceptible (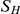), latently infected (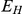), infectious (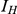) or recovered (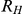). Two distinct infectious classes are defined. After the first infectious class a fraction of 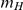 of the infectious hosts die from the disease. The remaining infectious hosts recover after the second infectious class. This model structure allows for the effect that a higher host mortality effectively reduces the average overall infectious period. A vector can only be susceptible (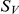), latently infected (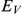, i.e. in the extrinsic incubation period) or infectious (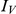); a recovered compartment is lacking because infectious vectors stay so for life.

For the hosts natural mortality is not taken into account, as the course of the infection is much shorter than the average life span of the host. For the vectors on the other hand, the natural mortality rate 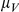 is much higher and should be included in the model. Assuming a constant hazard of dying, each vector has an equal probability of dying regardless of its age or class. The vector population is replenished by the birth of susceptible vectors. When the ratio between the number of vectors and the number of hosts varies over time, the birthrate is adjusted accordingly.

The virus can be transmitted from an infectious vector to a susceptible host. The rate of transmission depends on the number of infectious vectors 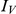 and the biting rate 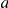, so 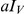 gives the number of infectious bites per day. The probability that an infectious vector bites a susceptible host is equal to the fraction susceptibles in the host population, 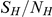, in which 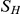 is the number of susceptible hosts and 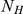 the total number of hosts. The probability that the bite of an infectious vector on a susceptible host is successful in transmitting the virus is 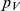. Thus, the transmission rate from vector to host is 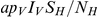.

The transmission rate from an infectious host to a susceptible vector is derived in a similar fashion. The number of susceptible vectors that bite per day is 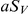, where 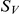 is the number of susceptible vectors. The probability that they bite an infectious host is the fraction 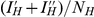, where 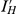 and 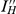 are the number of infectious hosts in the first and second infectious class. The probability that the virus is successfully transmitted by the bite of a susceptible vector on an infectious host is 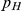. So, the transmission rate from host to vector is 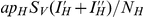.

With all the rates defined, a system of ordinary differential equations can be formulated. Here 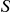, 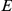, 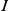 and 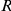 denote the number of susceptible, latently infected, infectious and recovered animals, and the subscripts 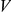 and 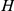 denote the vector and host. 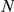 signifies the total number of vectors or hosts (i.e. 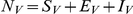 and 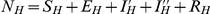). The vector compartments are described by:
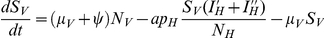


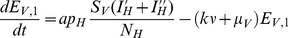
(1)

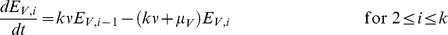


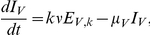
where 
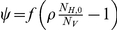
 is the rate at which the vector population adapts to changes in the vector to host ratio 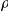, with 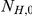 the number of hosts at the start of the epidemic. The vector to host ratio 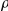 only changes in the simulations including seasonality, so 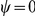 for the simulations without seasonality. The constant 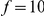 is sufficiently high for the vector population to follow the change in vector to host ratio but sufficiently low to avoid numerical issues. The host compartments are described by:
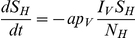


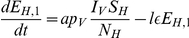


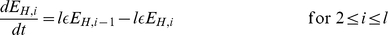


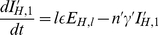
(2)

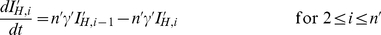


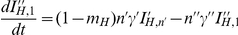


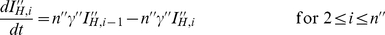


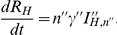



The average latent period is 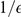 for hosts and the extrinsic incubation period is 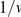 for vectors. The average infectious period for hosts is 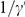 for the first infectious class and an additional 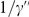 for the second infectious class. The latent vector compartment consists of 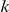 stages, the latent host compartment of 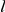 stages and the first and second infectious host class of 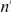 and 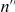 stages. The total number of infectious hosts in first and second infectious class 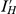 and 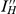 are consequently defined as 
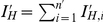
 and 
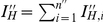
.

The biting rate 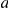, the extrinsic incubation rate 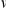 and the mortality rate 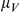 all depend on the temperature 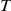 (in 
C). In warmer months the midges will bite more often, they will start transmitting virus earlier after infection, but they will also die sooner. From laboratory experiments the following empirical relations are derived [17], [18] (sections *S5*, *S6* and *S7* in Text S1):
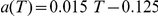


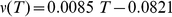
(3)

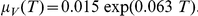



The solution of the ODE system (Equations 1–3) gives the course of the epidemic in the horse and midge populations. When the first infected horse (used as introduction source) has died or recovered, the number of infectious horses develops approximately exponentially. We will use this (fitted) exponential growth rate as a measure of how fast the epidemic progresses.

### Reproduction number

In epidemiology, transmission is often characterized by the basic reproduction number 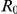, signifying the number of infections one infectious individual will cause during its entire infectious period in a fully susceptible population [19]. If 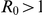 the virus can invade the population to cause an epidemic, while the infection will die out without affecting many hosts if 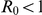. The reproduction number for a vector-borne disease includes the infection biology and demographics for both vector and host. It can be derived by considering the two transmission steps separately [16]. One infectious host will in a fully susceptible vector population infect on average 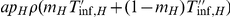 vectors, where 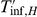 is the average infectious period of dying hosts (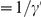) and 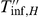 of recovering hosts (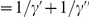). So, the term 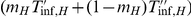 is the weighted average infectious period of the host. One infectious vector will in a fully susceptible host population infect on average 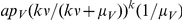 hosts, where the term 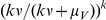 is the probability that a vector survives the extrinsic incubation period [16] and 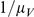 is the average life span of the vector. We will define the basic reproduction ratio 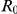 as the geometric mean of the two transmission steps:
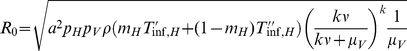
(4)


The reproduction number 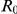 indicates whether a disease can initially spread through a population. However, this initial spread does not necessarily lead to an epidemic even though the reproduction number 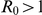. When examining the solution of the ODE system (Equations 1–3) the epidemic peak may fall so late that it would not qualify as an epidemic. Or - because of the deterministic solution - the number of infected vectors might drop below one or the peak in infectious hosts might not exceed one, at which points stochastic fade-out would be likely. So, to classify a simulation outcome as a local outbreak, we require that the host peak should be higher than one, that the infected vector population between the first and second generation should not drop below one and that the vector peak should be reached earlier than 365 days after introduction. Below we will introduce an additional requirement that transmission to other host herds occurs within 365 days to earn the classification of epidemic. This epidemic definition allows for a regression analysis to examine the effect of input parameters on the absence/presence of epidemics.

As some variables change periodically, such as temperature and vector density, a reproduction number with the threshold property at 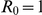 is not straightforward due to these seasonality effects [20]. Instead, the epidemic definition is used to examine the effect of the time of introduction on the fraction of introductions that will develop into an epidemic.

### Transmission to other host herds

When an outbreak unfolds in a group of horses, the neighbouring groups in the area are at risk of being infected as well. To protect these neighbouring groups and to apply control measures effectively, it is important to know how large the risk of spreading is and how fast the virus is expected to be transmitted. We will not consider the movement of horses as a transmission route because all transports are prohibited after the disease has been detected. Here we will focus on transmission through migrating vectors that are infected in the source herd. This is a best-case scenario that should be taken into account when discussing possible control measures for neighbouring groups.

The mobility of the vectors is often described as a diffusion process, where midges exhibit random flight behaviour. Assuming two-dimensional diffusion, the fraction of vectors 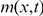 at time 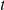 at distance 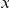 from the source is [21]:
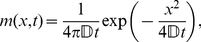
(5)where the diffusion coefficient 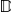 of *Culicoides* could be estimated from the capture-recapture experiments of Lillie et al. [22] (section *S13* in Text S1). However, midges are not continuously on the move but only when they actively search for a blood meal. Assuming an active search lasts one night, the probability of crossing the boundary with another group at distance 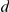 is 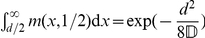. After infection the expected number of remaining active searches is the lifespan divided by the bloodfeeding interval or - equivalently - the biting rate divided by the mortality rate, i.e. 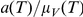. So, the expected fraction of vectors that migrate after infection is:
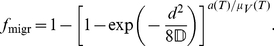
(6)


This fraction increases when groups of horses are closer together (smaller 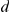), when the diffusion is higher (larger 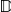) or at a higher temperature which increases the expected number of searches. To estimate the time at which the first successful transmission to other herds occurs, the expected number of successful transmissions to other herds 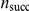 at time 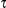 is calculated by:
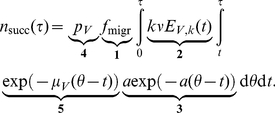
(7)


This expression can be broken down in different steps (denoted by numbers below Equation 7):

1. Of the infected vectors a fraction given by Equation 6 will migrate to another population and become infectious.
2. The number of infected vectors that become infectious at time 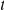 is given by the outflow of the last stage of the latently infected vector compartment, 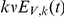.
3. After the vector becomes infectious at time 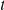 it can bite a host with the constant ‘hazard’ 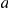. The probability that this happens at time 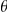 is exponentially distributed 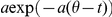,
4. with a chance of 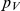 at being successful.
5. During the time until the vector bites a host, there is a continuous risk of the vector dying. So, the probability 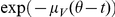 that the vector survives until time 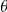 is taken into account.

All possible times of becoming infectious (at time 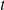) and biting (at time 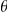) are covered by the double integral (with 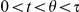). The inner integral is computed exactly as a function of the input parameters and times, after which it can be used for simple substitution to arrive at the result for the current parameters. The time 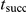 at which the first successful transmission to other herds is expected to occur, is found by solving 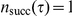 for 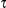. If 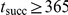 days, the outbreak is presumed not to spread to other populations, and the simulation is disqualified as epidemic (even when it does spread locally).

The speed of transmission to other herds, expressed by 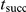, only estimates the first successful transmission to a susceptible host in the other herd. When more infected vectors migrate, the chance that the transmission to another herd will actually occur increases and the chance of multiple incursions increases, putting the neighbouring group at greater risk. We will use the number of successful transmissions to other herds 365 days after introduction in the source herd, 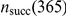, as a measure for this exposure risk to other populations.

### Model parameters

The model parameters are estimated from literature on laboratory experiments and field observations. It must be kept in mind that the vector species reported in literature do not occur in The Netherlands. As the number of donkeys in The Netherlands is relatively small, we will focus on horses as only host species. All model parameters are summarized in Table 1 and a full explanation of the chosen parameter values and ranges is provided in Text S1. Data on AHS in hosts and vectors is limited or non-existent, in which case data on bluetongue was used. Nonetheless, the variation of the parameter distributions is chosen to reflect this current knowledge.

Ten of the input parameters in Table 1 that are expected to be most influential, are varied in a range that characterizes the uncertainty and/or expected variation of a parameter value. To cover the entire parameter space, a collection of parameter values is generated by Latin Hypercube Sampling (LHS) [23]. Each of the distribution functions is divided in 100 equiprobable parameter values, that are combined randomly to yield 100 parameter sets. This is repeated 100 times, so in total 10 000 parameter sets are used in the LHS scheme. The use of 100×100 parameter sets allows for studying the distribution of model outcomes as a function of the input parameters. For each parameter set the ODE system (Equations 1–3) is solved numerically, starting with one infected horse in the first latent stage at time 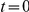. Five outcome variables are determined: epidemic absence/presence, reproduction number 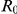, epidemic growth rate 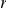, time of first successful transmission to other herds 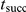 and exposure risk to other host groups 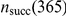.

The same parameter sets are used for an analysis of control effects. Five parameters are identified that could to some extent be controlled during an epidemic. The infectious period can be shortened when the infected host is euthanised after detection of clinical symptoms. Vaccination can also reduce the infectiousness (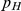) of infected hosts as well as the susceptibility (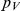) of non-infected hosts. Vector control would reduce the lifespan of midges (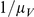) and there are several ways to reduce the biting rate (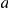). The degree of reduction in one or more parameter values that could be attributed to a particular control measure is unknown. For this reason we will vary the reduction of each of the controlled parameters between 0 (no effect) and 1 (full effect) and evaluate the impact on the five outcome variables.

For the model analysis without seasonality the conditions in August apply: the temperature and vector to host ratio do not change over time (although they differ in the various parameter sets). When seasonality is taken into account they will change during the year, fitted to long-term temperature measurements and *Culicoides* catch data (sections *S4* and *S10* in Text S1).

## Results

### Simulation example

**Figure 2:**
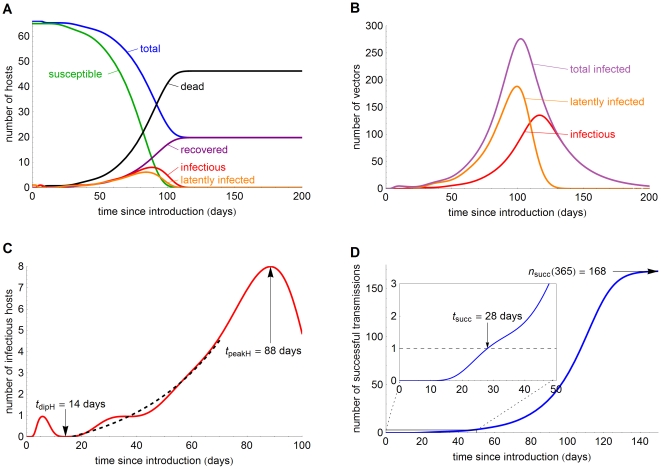
Dynamic behaviour using default parameter values. (a) the number of hosts (total 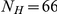), (b) the number of vectors (total 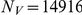), (c) the exponential function 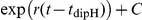 (dashed line) fitted to the number of infectious hosts and (d) the number of successful transmissions to other herds as a function of the time since introduction.

As an example we will examine the default parameter set (Table 1) that leads to the dynamic behaviour shown in Figures 2A and 2B. The reproduction number for these parameters is 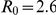 (Equation 4). The outbreak has at its peak 8.0 infectious hosts (more than one), the total number of infected vectors drops to 3.4 at 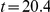 days (but not below one) and the infectious vector peak occurs at 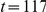 days (before 365 days after introduction). So according to our definition, this solution classifies as a local outbreak. Interesting to note here, is that all hosts are infected, while only a small proportion of the vectors is. At its peak only 276 of the originally 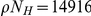 vectors are either in the extrinsic incubation period or infectious. The infectious vector peak occurs later than the infectious host peak due to the relatively long extrinsic incubation period of the infected vectors. After the first infected horse dies or recovers, the number of infectious hosts dips to almost zero at 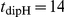 days and reaches its peak at 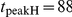 days (Figure 2C). To determine the exponential growth rate 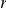 the function 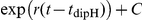 is fitted to the infectious host curve from 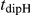 till 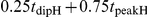, the upper limit being an arbitrary choice up to which exponential growth is assumed (dotted line in Figure 2C). In our example the fitted exponential growth rate is 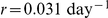. The number of successful transmissions to other herds 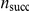 is monotonically increasing (Figure 2D), reaching unity at the expected time of the first successful transmission to other herds 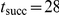 days. As this happens earlier than 365 days after introduction, this solution also classifies as an epidemic. The maximum at 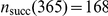 is used as measure of the exposure risk. Each of the 10000 parameter sets is analyzed in this way, to determine the effect of model parameters on the epidemic behaviour.

### Absence and presence of epidemics

**Figure 3:**
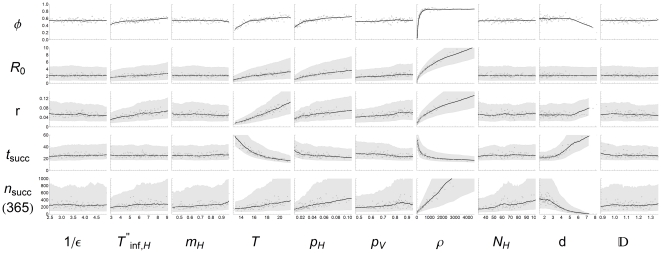
Results of uncertainty and sensitivity analysis. Effects of all input parameters (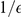: latent period of hosts (days), 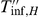: infectious period of recovering host (days), 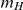: host mortality, 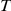: temperature (
C), 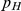: transmission probability from host to vector, 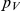: transmission probability from vector to host, 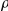: vector-to-host ratio, 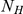: herd size, 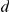: herd-to-herd distance, 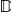: vector diffusion coefficient) on all model outcomes (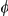: epidemic fraction, 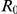: reproduction number, 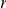: exponential growth rate, 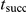: time of first successful transmission to other herds, 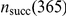: total number of successful transmissions to other herds). Dots indicate the average epidemic fraction (first row) or the median values of the continuous model outcomes (other rows), with the running average (solid line) and the 25%–75% interval for the continuous model outcomes (shaded area).

About 54% of the 10000 LHS parameter sets led to an epidemic. The first row of Figure 3 shows the fraction of simulations satisfying the epidemic definition as a function of all input parameters (see next section for the results in the remaining rows). Obviously, some parameters have little impact (such as the latent period of host) and others have a large effect (such as the temperature). To study the relative influence of input parameters on the absence and presence of epidemics, we use a logistic regression model. First the most parsimonious logistic model is determined by calculating the loglikelihood of the logistic regression model using all possible subsets of the ten input parameters. Comparison of the corresponding Bayesian information criterion [24] identified three parameters that can be eliminated from the model. The variation in the latent period of hosts only affects the generation time and does not influence whether an epidemic occurs or not. The herd size is eliminated from the logistic model as well. Naturally, a smaller population has a larger chance on stochastic fade-out, but this effect is not taken into account in the deterministic simulation model. Finally, also the vector diffusion coefficient can be discarded. Although this parameter can theoretically affect the transmission to other host herds, its variation is so small that the effect on the epidemic absence or presence is negligible compared to the effects of other parameters. In the resulting reduced model containing the remaining seven input parameters, two parameters have a negative estimate (Table 2). When the host mortality increases, the infectious period of hosts effectively reduces, thus lowering the chance on an epidemic. And when the distance between host herds increases, the chance of transmission to other herds also reduces. The deviance is a measure for the influence of each parameter on the absence and presence of epidemics. They are listed in order of decreasing deviance in Table 2. These show that the variation in vector to host ratio is the main determinant for the absence and presence of epidemics, as it affects both the local outbreak and the transmission to other host herds. The second most important determinant is the distance between host herds, that only influences the progression to other groups of horses. The temperature holds the third rank. The yearly temperature variation would most probably be more important, but this analysis only takes the temperature variation in August into account. The transmission probability from host to vector 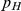 has a larger influence than that from vector to host 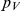, because the latter has a fairly high value and as a consequence its variation is relatively small.

**Table 2:** Results of the reduced logistic regression model on absence and presence of epidemics.

parameter	estimate	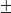	SE	z-statistic	p-value	deviance
intercept	−18.1	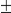	0.52	−34.5	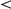 0.001	
logarithm of vector to host ratio 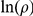	1.68	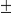	0.038	44.7	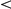 0.001	5986
herd-to-herd distance 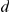	−0.586	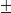	0.019	−31.5	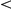 0.001	2045
temperature 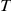	0.472	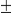	0.017	28.2	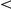 0.001	382
transmission probability from host to vector 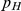	35.5	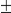	1.5	24.3	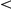 0.001	245
infectious period of recovering hosts 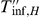	0.370	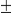	0.021	17.6	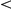 0.001	84
transmission probability from vector to host 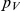	0.854	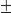	0.248	3.4	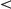 0.001	7
mortality hosts 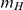	−0.936	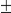	0.206	−4.5	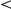 0.001	4
residual^a^						5049

Results of the reduced logistic regression model on absence and presence of epidemics, with SE the standard error.

a residual deviance on 9992 degrees of freedom.

**Figure 4:**
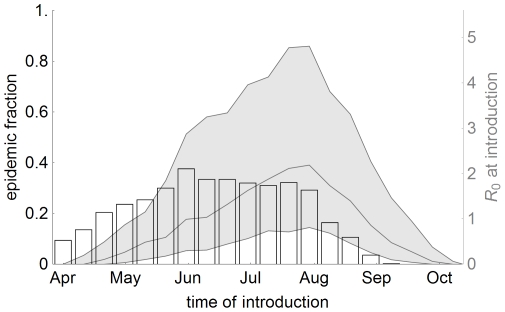
Effect of the time of introduction on the epidemic. The bars show the fraction of introductions that lead to an epidemic (left axis) and the shaded area denotes the median and 25%–75% interval of the reproduction number 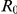 at the time of introduction (right axis).

As both the vector to host ratio and the temperature fluctuate during the year, we investigated the effect of the time of introduction on the absence and presence of epidemics. For this we added seasonality effects to the model (sections *S4* and *S10* in Text S1) and varied the introduction day between 100 and 300 (mid April to end October). The fraction of parameter sets that lead to an epidemic does not exceed 40% (Figure 4), which is considerably lower than the fraction of 54% for the simulations without seasonality. The maximum is reached from mid May to August, while the period when the median reproduction number 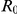 is above one falls later in the year, from June to September. This is because the reproduction number at the time of introduction does not take the ensuing conditions into account. So even though the reproduction number is below unity at the time of introduction, the conditions may improve and the increasing vector numbers and temperature will propel the early introduction into an epidemic.

### Uncertainty and sensitivity analysis

The effect of the input parameters on the other (continuous) model outcomes is studied in a sensitivity and uncertainty analysis. These effects are shown per input parameter and per outcome variable in Figure 3. For each model outcome, the slope shows the dependency on an input parameter and the variation reflects the effect of the other parameters, which together provides insight in the relative importance of the parameter. To compare the impact of the input parameters directly, we calculate the Kendall rank correlation coefficient (KRCC), a non-parametric statistic that determines the associations between input and outcome based on their ranks [25]. The KRCC is bound between 1 (perfect positive correlation) and −1 (perfect negative correlation). When the KRCC is (near) zero the compared quantities are not or only weakly correlated. For each collection of parameter sets the KRCC's between each input parameter and model outcome are calculated. With 100 collections of 100 parameter sets each, the median KRCC and its variation are determined. For the reproduction number 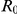 all 10000 parameter sets are used, while for the exponential growth rate and the transmission time and exposure to other groups only the parameter sets classified as epidemic are used.

**Figure 5:**
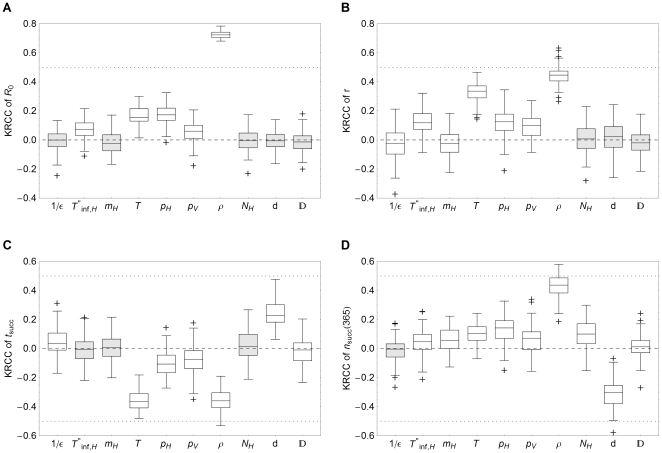
Kendall rank correlation coefficients of uncertainty and sensitivity analysis. Box and whisker plots of Kendall rank correlation coefficient (KRCC) between ten input parameters and (a) the reproductive number 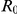, (b) the exponential growth rate 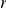, (c) the time 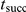 of successful transmission to other herds and (d) the number 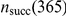 of successful transmissions to other herds. The input parameters are the latent period of hosts 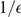, the infectious period of (recovering) hosts 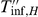, the host mortality 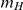, the temperature 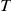, the transmission probability from host to vector 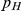, the transmission probability from vector to host 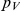, the vector to host ratio 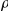, the herd size 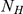, the herd-to-herd distance 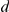 and the vector diffusion coefficient 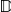. Boxes enclose the lower quartile, median and upper quartile, the whiskers indicate 1.5 times the interquartile range and crosses indicate outlying values. Mean coefficients that do not significantly differ from zero (p-value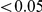) are shown in gray.

The KRCC results in Figure 5A show that the reproduction number 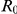 is not dependent on the latent period 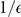, the herd size 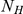, the distance between herds 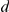 and the vector diffusion coefficient 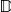, as these do not occur in the expression for the reproduction number (Equation 4). The host mortality 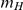 seems to have a slight negative correlation to the reproduction number 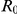, but this is not significant. For the remaining parameters, the KRCC results for 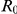 agree with the previous results of the logistic regression. The vector to host ratio 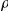 has the highest correlation to 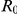, mainly due to the large expected variation in vector densities, and the infectious period 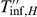, the temperature 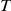 and the transmission probabilities 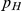 and 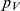 all have a positive correlation to 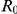. In all comparisons, 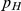 has a larger effect than 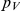 because of the small variation of the latter.

When an epidemic does occur, the exponential growth rate 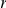 is important for (local) control purposes. The more slowly an epidemic develops, the more time is available for control measures to be implemented or to take effect. The KRCC's between the input parameters and exponential growth rate are calculated for the subset of epidemics. The results show that the most important determinants here are the temperature 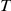 and the vector to host ratio 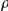 (Figure 5B). A longer latent period 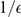 or a higher host mortality 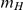 lead to slightly slower epidemics, but this effect is indiscernible compared to the other parameters (Figure 3). The infectious period 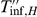 and the transmission probabilities 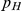 and 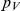 have comparable effects on the exponential growth rate. The exponential growth rate seems to be affected by the herd-to-herd distance too (Figure 3), caused by the effect that only local outbreaks with a high epidemic growth rate are able to bridge the larger herd-to-herd distances to succeed in transmission to other herds. This happens in too few epidemics to result in a significant effect (Figure 5B).

The interherd parameters 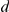 and 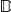 affect the transmission to other herds, characterized by the timing 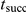 and the number 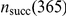 of successful transmissions to other herds (Figures 5C and 5D). The vector diffusion coefficient 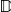 has a very weak correlation to the transmission to other herds, because of its small range. The herd-to-herd distance 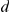 on the other hand greatly influences the transmission to other herds. At larger distances the time until the first successful transmission to other herds increases and the total exposure decreases. However, this effect is still smaller than the effect of the vector-to-host ratio 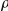, which is again the main determinant. Interestingly, the temperature 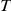 is very important for the time of transmission to other herds, but less so for the total exposure. The latent period 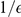 affects the time of transmission to other herds, but not the total exposure, whereas the opposite applies for the infectious period 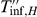, the host mortality 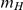 and the host herd size 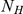. Also remarkable is that of the two transmission probabilities, 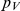 has the least impact on transmission to other herds, even though the timing and exposure explicitly depend on it (Equation 7).

### Effect of control measures

**Figure 6:**
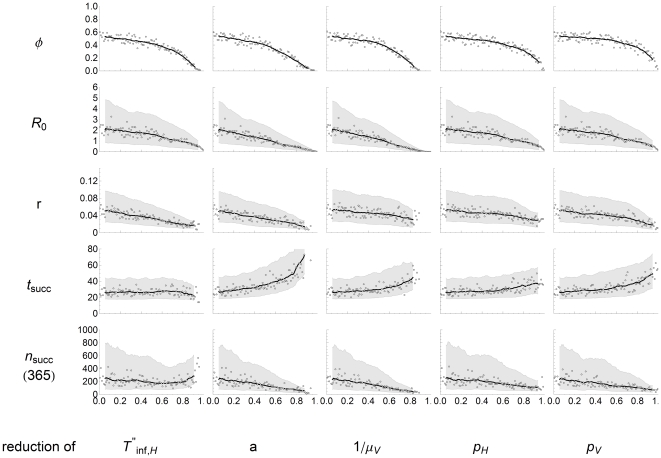
Results of control analysis. Effect of reduction of controlled parameters (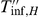: infectious period of recovering host (days), 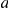: biting rate vectors, 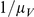: life span vectors, 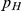: transmission probability from host to vector, 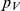: transmission probability from vector to host) on all model outcomes (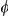: epidemic fraction, 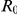: reproduction number, 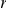: exponential growth rate, 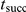: time of first successful transmission to other herds, 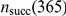: total number of successful transmissions to other herds). The controlled parameters are reduced with a factor between 0 (no effect) and 1 (full effect). Dots indicate the average epidemic fraction (first row) or the median values of the continuous model outcomes (other rows), with the running average (solid line) and the 25%–75% interval for the continuous model outcomes (shaded area).

**Figure 7:**
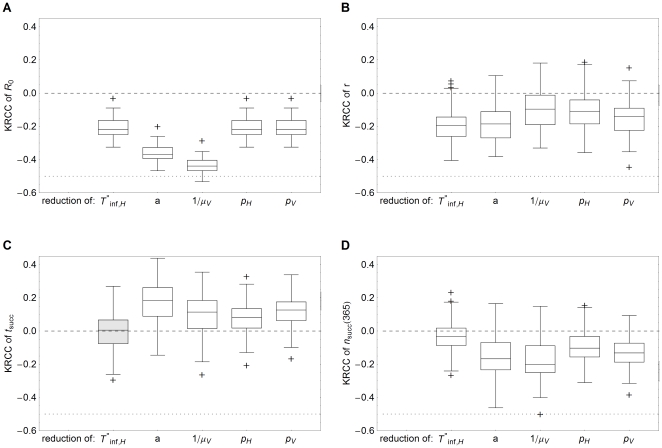
Kendall rank correlation coefficients of control analysis. Box and whisker plots of Kendall rank correlation coefficient (KRCC) between the reduction of five controlled parameters and (a) the reproductive number 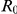, (b) the exponential growth rate 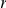, (c) the time 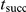 of successful transmission to other herds and (d) the number 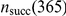 of successful transmissions to other herds. The controlled parameters are the infectious period of (recovering) hosts 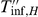, the biting rate 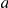 of vectors, the life span 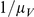 of vectors, the transmission probability from host to vector 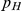 and the transmission probability from vector to host 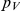. Boxes enclose the lower quartile, median and upper quartile, the whiskers indicate 1.5 times the interquartile range and crosses indicate outlying values. Mean coefficients that do not significantly differ from zero (p-value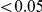) are shown in gray.

In the event of an AHS virus introduction in The Netherlands, several control measures can be taken, aimed at reducing the probability that the introduction develops into an epidemic, reducing the exponential growth rate and/or reducing the transmission to other herds. Five parameters are identified that can to some extent be controlled and the effect of reducing them is studied for these different model outcomes. Each control parameter is reduced separately by a factor between 0 (no effect) and 1 (full effect), but what reduction factor can be achieved in practice depends on the specific control measure. Similar to the uncertainty and sensitivity analysis, the effect per input/outcome pair and the KRCC are shown in Figures 6 and 7.

Reducing the infectious period of hosts can be achieved by euthanasia of infected hosts that show clinical signs. As these often show shortly after the host has become viraemic [26]–[28], immediate euthanasia can considerably reduce the infectious period (which would also increase the host mortality, but this is not taken into account here). A shorter infectious period mainly reduces the probability of an epidemic developing and the exponential growth rate.

The biting rate of vectors can be reduced by protecting a horse from midge bites in several ways. One option is to treat horses with insect repellents, but this has to be repeated often. Another option is to cover them with eczema blankets that are commonly used to protect sensitive horses from having an allergic reaction to midge bites (summer eczema or “sweet-itch”). However, the blanket does not cover the legs. A recent pilot study showed that even without a protective blanket, a considerable number of midges feed on the legs of the horse [29]. Finally, keeping the horse in its stable when midges are active is not expected to have a large effect, as catch data suggest that the vector numbers inside and outside are comparable (section *S10* in Text S1). Only when stables have proper vector protection, such as meshing impregnated with insecticides, midge numbers inside can decrease. All these measures discussed here will reduce the biting rate, but it can not be expected that they will protect horses completely. Nonetheless, reducing the biting rate has a beneficial effect on all model outcomes. The probability of an epidemic is lowered, the exponential growth rate reduced, the transmission time to other herds is most effectively reduced and the total exposure to other herds decreases as well.

Reducing the life span of *Culicoides* can be achieved by vector control. This measure is not aimed at decreasing the vector population (that is unaltered in the simulations), but it gives infected midges less chance to survive their extrinsic incubation period. So, not the entire vector population needs to be covered, but focusing on infected midges is sufficient, for instance in a stable with (possibly) infected horses. This has a large impact on the probability of an epidemic and the reproduction number, but - when an epidemic does occur - less on the exponential growth rate and on the transmission to other herds.

Vaccination triggers the horse's immune system to develop antibodies against the AHS virus. This has the combined effect of reducing both transmission probabilities. Vaccination challenge studies show that vaccinated horses can be protected from infection [26]–[28]. This lower susceptibility can be interpreted as a lower transmission probability from vector to host 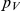, even though a direct challenge is not equivalent to an infectious midge bite. When a (partly) vaccinated horse does get infected, viraemia levels are typically lower in vaccinated animals than in non-vaccinated animals [28], likely to result in a lower transmission probability from host to vector 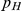. Both reduced transmission probabilities lead to beneficial albeit modest effects on the model outcomes. Reducing the transmission probability from vector to host 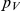 has the larger effect, and high reduction factors are achievable (approaching 1 when a host if fully protected). So, we expect that vaccination is mainly effective by protecting vaccinated animals from infection, provided that the immune system has sufficient time to respond. Vaccination studies show elevated titres from 3 to 4 weeks after the first vaccination [27], which is longer than the extrinsic incubation period of midges and the time scale of transmission to other herds. Herds in direct vicinity of the source herd will therefore probably not benefit from vaccination in time, especially as migration of infected vectors may not be the only transmission route. When hosts are not fully protected by vaccination, they might be infected without showing clinical signs. In this case transmission might still occur, and they might be infectious for a prolonged period [28]. For this reason, we argue that vaccination is used to protect susceptible herds, rather than to lower the infectiousness of infected herds. In practice we would recommend that herds farther away are prioritized for vaccination over herds in direct vicinity of the infection source.

## Discussion

The model presented in this paper integrates the current knowledge on the virus, the vector and the host of African horse sickness. By allowing additional stages for the latent and infectious compartments, it resembles the infection biology as closely as possible. The distributions of the input parameters are chosen either to take the uncertainty into account or to cover the range of conditions encountered in The Netherlands. By studying relevant model outcomes we have identified the most important parameters that drive an epidemic and assessed the effect of control measures.

In our model description we have included only one vector species and one host species. We have implicitly assumed that all Dutch *Culicoides* species are competent vectors. It is generally thought that the competent vectors for AHS and bluetongue transmission coincide because of the virus similarity. As the *C. obsoletus* complex - the most common *Culicoides* species complex in The Netherlands - is indicated as a competent vector for bluetongue transmission, we have assumed that this would also be the competent vector for AHS transmission. In our model analysis we have ignored donkeys as host species, because of their relatively low numbers in The Netherlands and because an epidemic cannot be sustained in donkey populations [1]. However, they can play a role in virus introduction, because of their long infectious period and lack of clinical signs.

The reproduction number 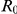 is related to the probability that a virus introduction develops into an epidemic. Our results show that the vector-to-host ratio has the largest impact on both the reproduction number and the absence/presence of epidemis. This agrees with the results of Lord et al. [7] who used a model with explicit seasonality in the vector population. Gubbins et al. [30] however, identified the temperature as most influential (for bluetongue). The difference is probably explained by the extended, uniformly distributed temperature range (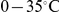), while our study was restricted to normally distributed full day temperatures in the month of August (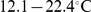). As the vector-to-host ratio differs per region (depending on vegetation, soil, humidity, etc.), the reproduction number can be used for risk maps, to assess whether a virus introduction would be able to cause an AHS epidemic in different areas in The Netherlands. As both the temperature and vector-to-host ratio fluctuate during the year, risk maps can also be produced for different periods [31]. However, the reproduction number at the time of introduction poorly correlates to the establishment of an epidemic, so this should be kept in mind when interpreting this kind of risk maps.

When an epidemic does occur, it is important to know the exponential growth rate and the timing and risk of transmission to other herds. Different input parameters have different effects on these quantities. Besides the vector-to-host ratio, the temperature becomes important for the transmission locally and to other herds, and the distance between host herds largely determines the timing and risk of transmission to other herds. So, a high temperature accelerates the epidemic, while a high horse density increases the extent of the epidemic.

Because of the similarity in virus and vector, it is interesting to consider the analogous case of bluetongue, that has proved to spread effectively in temperate regions of Northwestern Europe [3], [4]. Compared to bluetongue, AHS has three notable differences. First, AHS infected horses are infectious for approximately 1 week (Table 1), while bluetongue infected sheep and cattle are viraemic for 2 and 3 weeks [30]. This means an AHS epidemic will develop more slowly, and the probability of the virus surviving the winter in an infectious host is smaller. Secondly, AHS infected horses will show more apparent clinical signs, which means they can be fast and effectively detected, and removed if necessary. During the bluetongue epidemic in 2006 infected farms were reported 12–17 days after the onset of clinical signs [4]. And finally, of the hosts on which midges feed, a large fraction is susceptible to bluetongue (all ruminants), and only a small fraction to AHS (all equines). In mixed herds or horse herds interspersed with cattle or sheep herds, the chances that a midge bites an infectious host and the chance that infectious midge bites a susceptible host are smaller. The biting rate on horses effectively reduces because of the diluting effect of the non-susceptible hosts. For these reasons, we expect AHS to invade and spread less easily than bluetongue.

Using the diluting effect of non-susceptible hosts as an an active control measure to reduce the biting rate, seems an attractive option. It could be effective when two assumptions were true. First, the vector to host ratio should be unaltered (i.e. when our horses are joined by an equal number of sheep, the vector population doubles) or lowered. And secondly, the vectors transmitting the disease should not have a host preference or they should prefer the non-susceptible hosts. However, both of these assumptions are uncertain. It might be possible that the non-susceptible hosts attract more than a proportional number of vectors, or provide better breeding sites (e.g. in cow manure). When these vector species have a higher preference for horses (e.g. due to a thinner skin), they will feed primarily on the available horses. All these effects will increase instead of decrease the number of bites on horses, amplifying the epidemic. More research is needed to study the midge attraction and host preference, before it can be concluded whether this dilution method would work or not. For the present it is not recommended to add or remove non-susceptible hosts. In this way it will be prevented that they would transport possibly infected midges.

In conclusion, transmission of AHS is strongly determined by the time of introduction and the associated temperature and vector season. The vector density is a more important indicator for establishment and spread than the host density. In areas with a low horse density, the virus will not be able to spread to a far-away neighbouring herd. And in areas with a high horse density, the probable presence of non-susceptible hosts will moderate the transmission of AHS. Due to the severe and fast clinical signs, early detection of AHS is possible and control should focus on a fast repression of the virus spread. We argue that this could be achieved by the following control measures. Horses with severe clinical signs should be euthanized, for the removal of an infection source as well as for welfare reasons. The infected herd should be kept in a stable, if possible with protective meshing, where active vector control should prevent infected midges from escaping. In neighbouring herds control should focus on reducing midge bites, either by protective blankets, insect repellant or shielded stables. In herds farther away, vaccination can protect horses from infection, provided that a safe and effective vaccine is available. To reduce spatial spread and to give vaccination sufficient time to be effective, transport regulations should be strictly applied and maintained. For a better understanding of AHS outbreaks in temperate regions, it is recommended to study the competence and host preference of the different *Culicoides* species present.

## Supporting Information

Text S1 **Parameter estimation.** Parameter estimations for latent period in hosts, infectious period in hosts, disease-induced mortality in hosts, temperature, biting rate, extrinsic incubation rate, vector mortality rate, transmission probability from host to vector, transmission probability from vector to host, vector to host ratio, host herd size, host herd distance, and vector diffusion coefficient.
(PDF)
