# Supplementary material for: Transmission and Control of African Horse Sickness in The Netherlands: A Model Analysis
Source: PLoS One. 2011 Aug 5;6(8):e23066. doi: 10.1371/journal.pone.0023066 (PMC3151287; doi:10.1371/journal.pone.0023066)
Supplement: Text S1 — Parameter estimation. Parameter estimations for latent period in hosts, infectious period in hosts, disease-induced mortality in hosts, temperature, biting rate, extrinsic incubation rate, vector mortality rate, transmission probability from host to vector, transmission probability from vector to host, vector to host ratio, host herd size, host herd distance, and vector diffusion coefficient. (PDF) [file pone.0023066.s001.pdf]

Parameter estimation for

Transmission and control of African horse sickness in The Netherlands: a model analysis

Jantien A. Backer[a,*], Gonnie Nodelijk[a]

[a]*Department of Epidemiology, Crisis Organisation & Diagnostics, Central Veterinary Institute of Wageningen UR, Lelystad, The Netherlands*

Model parameters

The parameterization of the model depends on literature of experiments that were not carried out in The Netherlands. Most of the experiments were done in South Africa with local breeds of horses. The virus strains used were mainly AHSV-4, AHSV-5 and AHSV-6, for the other 6 strains little experimental results were found. The studied vector species were either field caught (mainly *C. imicola* and *C. bolitinos*) or laboratory bred (*C. sonorensis*). None of these are present in The Netherlands, where most *Culicoides* are of the Obsoletus and Pulicaris complexes.

S1. Latent period in hosts.

To estimate the latent period of infected hosts the experimental results were used from five unvaccinated challenged horses that served as controls in vaccination-challenge studies [3, 11, 12]. These five horses were first viraemic at 3, 4, 4, 4, and 6 dpi, which - with a sampling regime of once a day - leads to estimated latent periods of 2.5, 3.5, 3.5, 3.5 and 5.5 days. Using these data the latent period was estimated as a gamma distibution with a mean of 3.7 days and a standard deviation of 0.9 days (corresponding to 16 latent stages, Fig. A1). The variation of the mean latent period is modelled by a normal distribution with a mean of 3.7 days and a standard deviation of 0.85 days, i.e. a 5%-95% interval of 2.5-4.9 days.

The control horses in these experiments were challenged by inoculation rather than by the bite of an infectious vector, which could affect the length of the latent period. However, experiments with bluetongue virus have shown that the latent period in sheep is similar after inoculation [5] and natural infection [1]. Therefore, we will assume that the infection method does not affect the latent period in hosts.

*Corresponding author
Email address: `jantien.backer@wur.nl` (Jantien A. Backer)

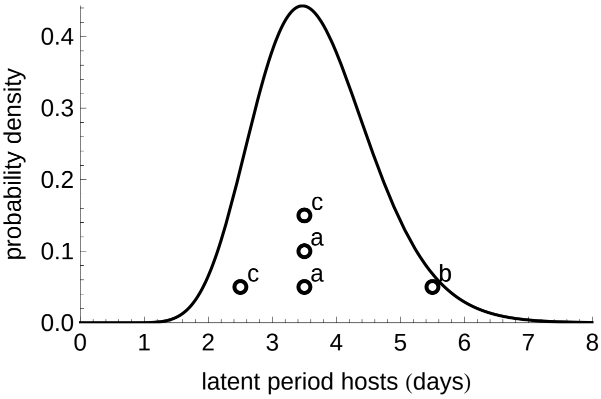

Figure A1: Latent period distribution of infected horses (solid line) fitted to experimental data (open circles) of (a) House et al. [3], (b) Roy et al. [11] and (c) Scanlen et al. [12] as a function of days post inoculation (dpi).

S2. Infectious period in hosts.

Five unvaccinated challenged horses that served as controls in vaccination-challenge studies [3, 11, 12] were viraemic in blood for 3, 4, 4, 5 and 6 days, after which all died. Assuming a direct relation between viraemia in blood and infectiousness, we estimated the infectious period distribution from these data. Fitting a gamma distribution yielded an infectious period for dying hosts with a mean of 4.4 days and a standard deviation of 1.0 days (corresponding to 19 infectious stages for dying hosts, Fig. A2).

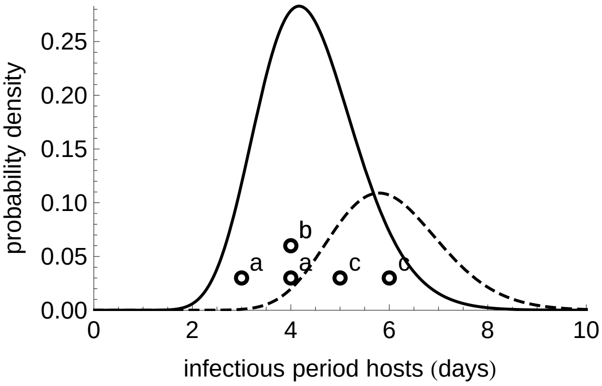

Figure A2: Infectious period distribution of infected horses, of which 70% die (solid line) and 30% recover (dashed line). The infectious period of dying horses is fitted to experimental data (open circles) of (a) House et al. [3], (b) Roy et al. [11] and (c) Scanlen et al. [12].

In general, recovering hosts are expected to have a slightly longer infectious period. As no experimental data were available we used the literature value of 6 (4 - 8) days reported by Mellor and Hamblin [8]. A gamma-distributed infectious period with a mean of 6 days and a standard deviation of 1.1 days was used (corresponding to 29 infectious stages for recovering hosts, or - almost equivalently - 10 extra infectious stages with a total retention time of 1.6 days after the 19 infectious stages for dying hosts, Fig. A2). Throughout the analyses, the mean infectious period of dying hosts will be fixed at 73% of the mean infectious period of recovering hosts. The variation of this latter mean infectious period is

modelled by a normal distribution with a mean of 6 days and a standard deviation of 1.4 days, i.e. a 5%-95% interval of 3.0-9.0 days.

S3. Disease-induced mortality in hosts.

African horse sickness can cause four forms of disease that differ in clinical signs and mortality [8]. Horse sickness fever is the least severe form without any mortality and is said to be exhibited by African donkeys and zebras only. The cardiac form can cause a mortality of 50% while the - most severe - pulmonary form causes death in 95% of the cases. The mixed form, a combination of cardiac and pulmonary form, has an average mortality of 70%. Although this mixed form and associated mortality is the most common in African horse populations, it is expected that the mortality in the naive Dutch horse population will be greater. Nevertheless, we will choose a mortality of 70% as default value. This can be considered as a 'worst-case scenario' as the average infectious period will be longer with a lower mortality (remember that recovering hosts have a longer infectious period than dying hosts). The host mortality will be uniformly varied between 40% and 100%.

S4. Temperature.

The temperature distributions were taken from monthly averages during the period 1971-2000 from weather station De Bilt [4]. For each month a normal distribution was fitted to the variation of the mean temperature, assuming that the reported interval between lowest and highest mean temperature represented the interval between the 1st and 99th percentile. The temperature variation over the year is used in the model analysis with seasonality (Fig. A3), while the analysis without seasonality uses the temperature distribution in August (inset Fig. A3). The mean temperature in August, 17.2°C, is used as default temperature.

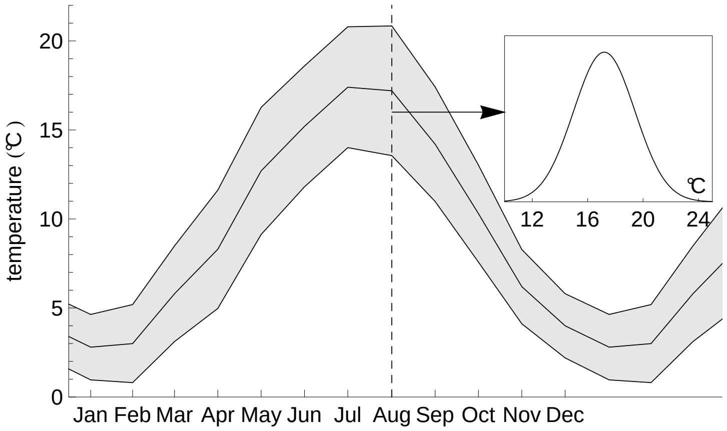

Figure A3: Monthly temperature variation and the temperature distribution of August (inset); the shaded area indicates the 5th percentile, median and 95th percentile.

S5. Biting rate.

Only female *Culicoides* need blood meals from hosts for egg development. Mullens and Holbrook [9] determined the mean period between blood feeding and oviposition as a function of temperature. As midges can take a next blood meal on the same day as oviposition, this period can be considered as the mean blood feeding interval. In general this blood feeding interval decreases with increasing temperature, due to the shorter gonotrophic cycle at higher temperatures. In contrast to Wittmann et al. [16], we fitted the biting rate a (which is the reciprocal of the blood feeding interval) to the experimental data, as a linear function of the temperature T (in °C, Fig. A4):

$$a(T) = 0.015T - 0.125. \tag{1}$$

At the default temperature of 17.2°C the blood feeding interval is 7.5 days. Mullens and Holbrook [9] used the same data to determine a different temperature-dependent function, but the values of both functions are similar within the measured range.

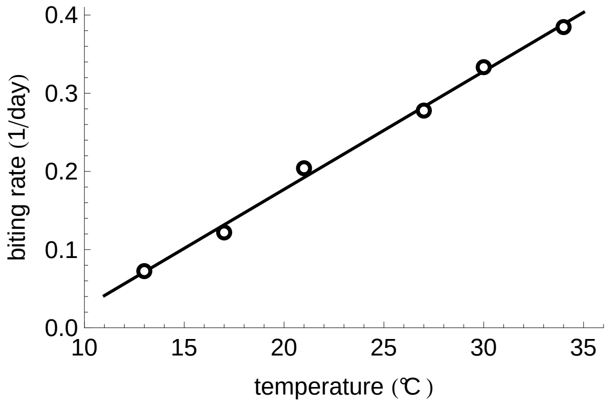

Figure A4: Biting rate (day^{-1}) of *C. variipennis* as a function of temperature; fit (solid line) to experimental data (open symbols) of Mullens and Holbrook [9].

S6. Extrinsic incubation rate.

After feeding on an infectious host, the viral titres of the vectors decrease to zero in a few days, but they start to increase again when the virus replicates in the vector. After this extrinsic incubation period they are infectious to susceptible hosts. In general, the extrinsic incubation period decreases with temperature, due to faster virus replication. Wittmann et al. [16] determined the extrinsic incubation rate (which is the reciprocal extrinsic incubation period) for AHSV serotype 4 in *C. sonorensis* experimentally as a function of the temperature. The extrinsic incubation rate ν depends linearly on the temperature T (in °C, Fig. A5):

$$\nu(T) = 0.0085T - 0.0821. \tag{2}$$

At the default temperature of 17.2°C the incubation period is 16 days. The temperature-dependent extrinsic incubation rate of bluetongue determined by Gerry and Mullens [2] differs in shape but the values are surprisingly similar. For the extrinsic incubation period of infected vectors we choose a gamma distribution with 10 stages. This is a fairly arbitrary choice due to lack of data.

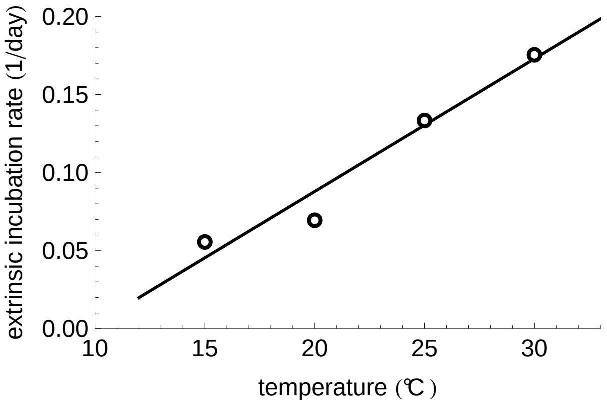

Figure A5: Extrinsic incubation rate (day^{-1}) of AHSV serotype 4 in *C. sonorensis* as a function of temperature; fit (solid line) to experimental data (open symbols) of Wittmann et al. [16].

S7. Vector mortality rate.

Midges are relatively short-lived insects and at higher temperatures their life span decreases even further. Wittmann et al. [16] determined the mean survival of *Culicoides sonorensis* at different temperatures and at different relative humidities. We fitted an exponential relationship between the mortality rate μ_V (which is the reciprocal life span) and the temperature T (in °C) to the experimental data at 75% relative humidity (Fig. A6):

$$\mu_V(T) = 0.015 \exp(0.063T). \tag{3}$$

At the default temperature of 17.2°C the average life span is 22 days. It is assumed that the hazard of dying is constant, which means the life span is exponentially distributed. This is confirmed by experimental survival rates of approximately 50% after 10 days [10, 14]. Infection with AHS does not influence the mortality rate of midges.

Gerry and Mullens [2] determined the vector mortality for midges in the field and found significantly higher values, which would lead to a reduced transmission. Nonetheless, we will use the vector mortality based on the data of Wittmann et al. [16], as a conservative choice and because these are the same experiments in which the extrinsic incubation period for AHSV was determined.

S8. Transmission probability from host to vector.

The probability that a vector is infected by biting an infectious host, can be studied by feeding a large number of midges on AHS virus containing blood through a skin membrane. After 10 days of

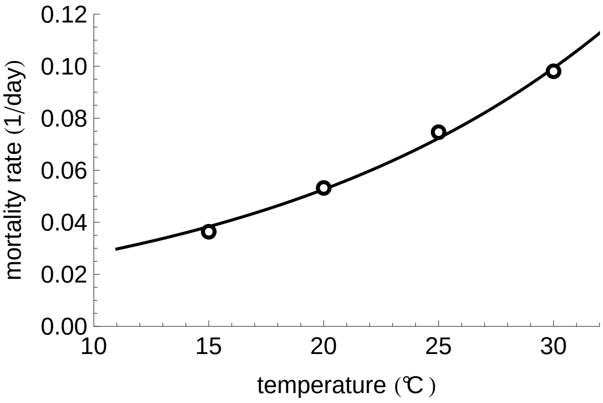

Figure A6: Mortality rate (day^{-1}) of *C. sonorensis* as a function of temperature; fit (solid line) to experimental data (open symbols) of Wittmann et al. [16] at 75% relative humidity.

extrinsic incubation Paweska et al. [10] recovered AHS virus serotype 7 (vaccine-)strains from 102/2338 (4.4%) midges of different species and Venter and Paweska [14] recovered different AHS virus strains from 68/1749 (3.9%) midges of different species. Even though the results of both experiments are very similar, for midges feeding on bluetongue-infected blood a much wider range of infection probabilities is reported [15, 1]. For this reason, the variation of the host to vector transmission probability is modelled by a gamma distribution with a median of 0.04 and a standard deviation of 0.03, i.e. a 5%-95% interval of 0.01-0.10.

S9. Transmission probability from vector to host.

No experimental results for AHS could be found in literature to determine the probability that a host is infected by the bite an infectious vector. It is generally thought that the transmission probability from vector to host is much higher than from host to vector. This idea is confirmed by a recent bluetongue experiment [1], where a single infectious midge was brought into contact with one susceptible sheep. Of the six replicate experiments, five showed a successful transmission. We will use this result to model the transmission probability from vector to host as a beta distribution, Beta(5+1,6-5+1), following the rule of succession. The median of this distribution is 0.77, that will serve as default value. The corresponding 5%-95% interval is 0.48-0.95.

S10. Vector to host ratio.

The numbers of vectors that feed on one host are heavily fluctuating and depend on the season, temperature, soil, wind conditions, rainfall, etc. During 2007 the vector numbers were determined every week on 20 different locations in The Netherlands, both inside and outside livestock barns (Fig. A7), resulting in 1227 trap collections, of which 776 included various species of *Culicoides*. As the vector numbers caught inside and outside do not differ markedly, the data were combined (Fig. A8). In the periods January-March and November-December virtually no *Culicoides* were caught, while in the period April-October they were trapped in largely varying numbers. When assuming that one light trap mimics

the attraction of one host on *Culicoides*, we can translate trap data directly to vector to host ratios. We will use the catches per month to fit a Weibull distribution (Fig. A8). For the September data an average of 513 midges per trap is fitted, which reasonably agrees with the mean number of 333 per trap found by Meiswinkel et al. [7] in the same month. For the model analysis without seasonality the August data apply, with a median of 226 vectors per host that will be used as default value. The corresponding 5%-95% interval is 1-4219 vector per host.

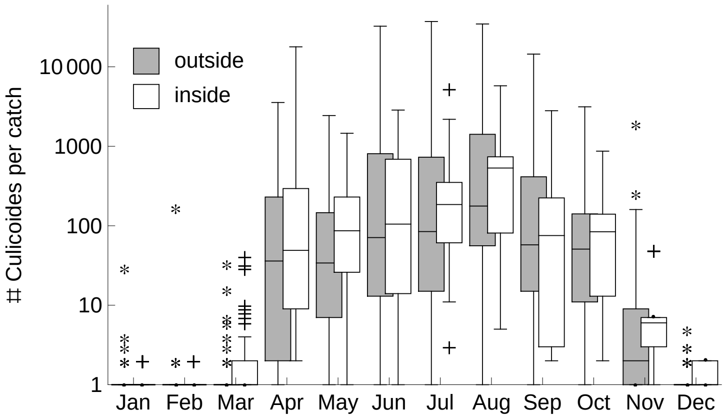

Figure A7: Box and whisker plots of the number of *Culicoides* per trap, caught inside (white) and outside (gray) livestock barns, per month in 2007 (boxes enclose the lower quartile, median and upper quartile, the whiskers indicate 1.5 times the interquartile range and crosses indicate outlying values).

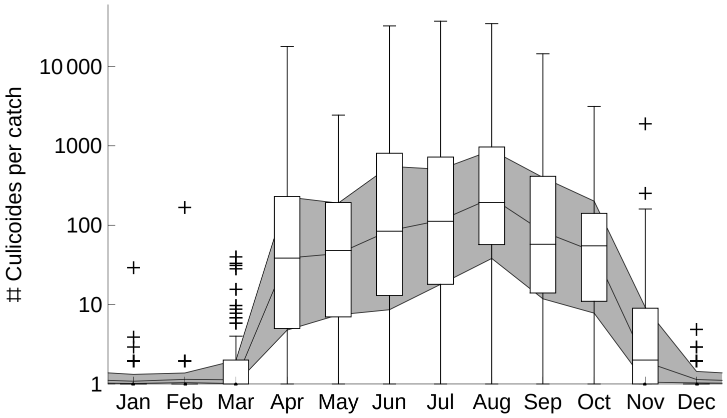

Figure A8: Box and whisker plots of the number of *Culicoides* per trap, per month in 2007 (boxes enclose the lower quartile, median and upper quartile, the whiskers indicate 1.5 times the interquartile range and crosses indicate outlying values). The shaded area encloses the lower quartile, median and upper quartile of the fitted Weibull distributions.

S11. Host herd size.

The total horse population in The Netherlands is estimated to be 450 000 animals, which are kept in small groups varying from a few in pastures up to one hundred in commercial stables. In contrast to livestock animals, central registration of horses and their locations is not obligatory. Instead, a simple

internet search with various keywords resulted in 6834 locations where horses are kept, leading to an average herd size of 66 horses. This population can be interpreted as a range of pastures with horses interconnected by a mobile population of midges, or as a reasonably sized commercial stable. The variation of the herd size is modelled by a normal distribution with a mean of 66 horses and a standard deviation of 21 horses, i.e. the 5%-95% interval is 32-100 horses.

S12. Host herd distance.

The 6834 holdings that were found in the internet search, were used to estimate the herd density in The Netherlands. This density seems to be exponentially distributed (Fig. A9) with an average density of 0.19 herds/km^2 or equivalently 5.2 km^2 per herd. This distribution $\text{pdf}_x(x) = \lambda\exp(-\lambda x)$ is transformed to the probability distribution function $\text{pdf}(d)$ for the farm-to-farm distance d, by solving $\text{pdf}(d)\text{d}d = |\text{pdf}_x(x)\text{d}x|$ and substituting $x(d) = 2/(\sqrt{3}d^2)$. The latter relation assumes that each farm occupies an area of $\frac{1}{2}\sqrt{3}d^2$ (based on hexagonal tiling, Fig. A10):

$$\text{pdf}(d) = \frac{4\lambda\exp(-\frac{2\lambda}{\sqrt{3}d^2})}{\sqrt{3}d^3}, \tag{4}$$

with $\lambda = 5.2$ km^2/herd. The average farm-to-farm distance is 2.4 km, and the 5%-95% interval is 1.4-10.8 km (inset Fig. A9).

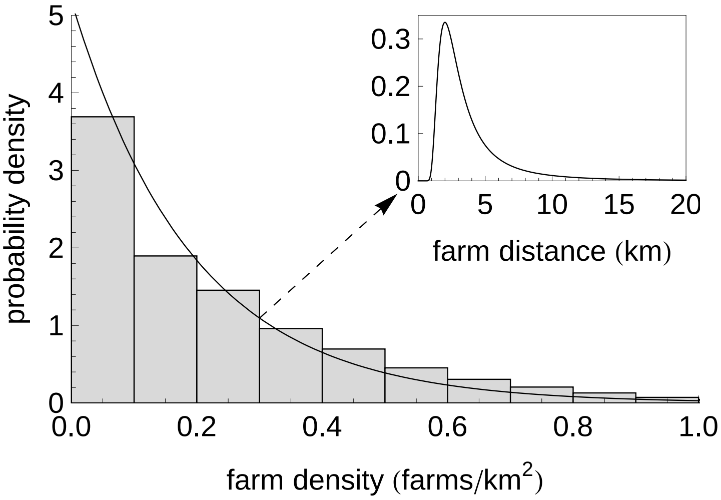

Figure A9: Farm density distribution for (estimated) locations of horse holdings in The Netherlands (gray bars) and estimated exponential distribution (solid line). Inset shows the resulting farm-to-farm distance distribution.

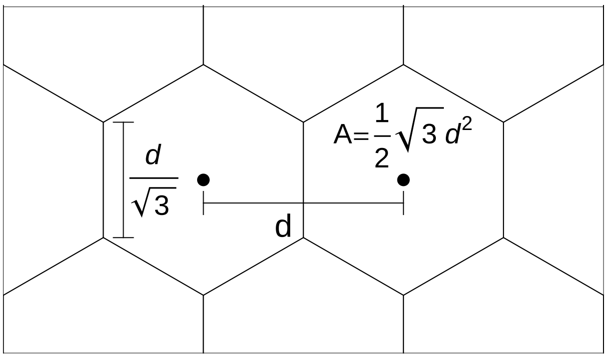

Figure A10: Assumed hexagonal tiling for horse holdings with interfarm distance d, occupying an area of $\frac{1}{2}\sqrt{3}d^2$.

S13. Vector diffusion coefficient.

The connectivity between neighbouring herds depends on the herd-to-herd distance d and the mobility of the vectors. The latter is often described as a diffusion process, where midges exhibit random flight behaviour. Assuming two-dimensional diffusion, the fraction of vectors $m(x,t)$ at time t at distance x from the source is [13]:

$$m(x,t) = \frac{1}{4\pi \mathbb{D} t} \exp\left(-\frac{x^2}{4\mathbb{D}t}\right), \tag{5}$$

with $\mathbb{D}$ the diffusion coefficient of *Culicoides*. The fraction of midges found between an inner radius x_{in} and an outer radius x_{out} at time t is $\int_{x_{\text{in}}}^{x_{\text{out}}} m(x,t)\mathrm{d}x = \exp\left(-\frac{x_{\text{in}}^2}{4\mathbb{D}t}\right) - \exp\left(-\frac{x_{\text{out}}^2}{4\mathbb{D}t}\right)$. We used this fraction and the capture-recapture experiments of Lillie et al. [6] to estimate the diffusion coefficient $\mathbb{D}$. They released 82200 midges and recaptured 380 during three consecutive nights using 7 traps up to 4 km from the release point. We assume that the midges are only active for half of the day, starting at release, giving the three consecutive catching nights the time stamps of 0.25 day, 1.25 days and 2.25 days post release. The maximum likelihood estimation resulted in $\mathbb{D} = 1.12$ (0.89-1.44) km^2/day, with the 95% confidence interval between brackets. Figure A11 shows the observed and estimated fraction of recaptured midges for three consecutive nights. The model fits the data poorly on the last night (Figure A11c), but its effect on the estimated diffusion coefficient is small as these data represent only 12 out of 380 caught midges that were used for the estimation. The variation of the vector diffusion coefficient is modelled by a normal distribution with a median of 1.12 km^2/day and a standard deviation of 0.14 km^2/day, i.e. a 5%-95% interval of 0.89-1.36 km^2/day.

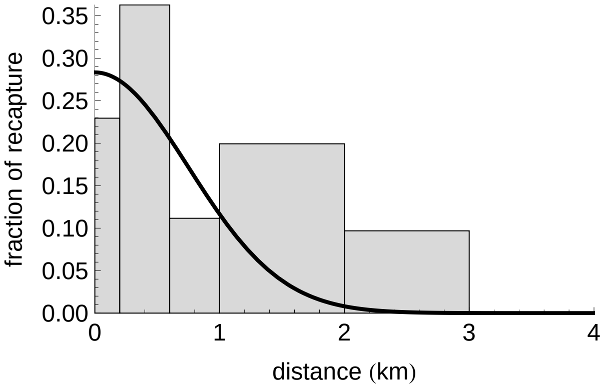

(a) release night (t=0.25 day)

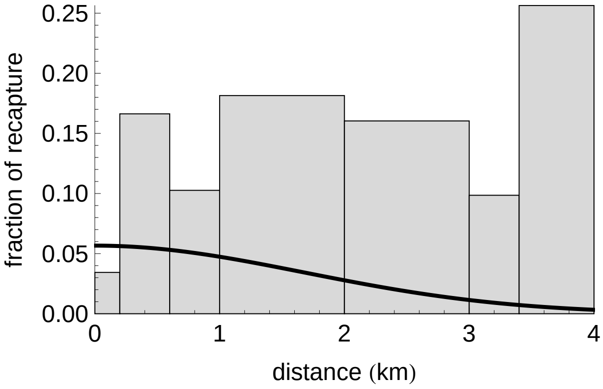

(b) one night post release (t=1.25 day)

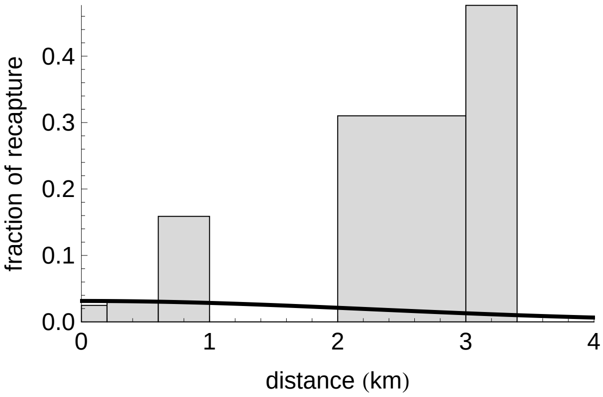

(c) two nights post release (t=2.25 day)

Figure A11: Observed recaptured fractions (gray bars) and estimated recaptured fraction (solid line) for (a) the release night, (b) one night post release and (c) two nights post release.

References

[1] Baylis, M., O'Connell, L., Mellor, P. S., 2009. Rates of bluetongue virus transmission between *Culicoides sonorensis* and sheep. Medical and Veterinary Entomology 22, 228–237.

[2] Gerry, A. C., Mullens, B. A., 2000. Seasonal abundance and survivorship of Culicoides sonorensis (Diptera: Ceratopogonidae) at a southern California dairy, with reference to potential bluetongue virus transmission and persistence. J. Med. Entomol. 37, 675–688.

[3] House, J. A., Thomas, M., Dubourget, P., House, C., Mebus, C. A., 1994. Further studies on the efficacy of an inactivated African horse sickness serotype 4 vaccine. Vaccine 12, 142–144.

[4] KNMI, 2009. Long-term weather averages 1971-2000. `http://www.knmi.nl/klimatologie/normalen1971-2000/station_pdf.html`.

[5] Koumbati, M., Mangana, O., Nomikou, K., Mellor, P. S., Papadopoulos, O., 1999. Duration of bluetongue viraemia and serological responses in experimentally infected european breeds of sheep and goats. Veterinary Microbiology 64, 277–285.

[6] Lillie, T. H., Marquardt, W. C., Jones, R. H., 1981. The flight range of *Culicoides variipennis* (Diptera: Ceratopogonidae). The Canadian Entomologist 113, 419–426.

[7] Meiswinkel, R., Goffredo, M., Leijs, P., Conte, A., 2008. The *Culicoides* snapshot: A novel approach used to assess vector densities widely and rapidly during the 2006 outbreak of bluetongue (BT) in The Netherlands. Prev. Vet. Med. 87, 98–118.

[8] Mellor, P. S., Hamblin, C., 2004. African horse sickness. Vet. Res. 35, 445–466, (doi:10.1051/vetres:2004021).

[9] Mullens, B. A., Holbrook, F. R., 1991. Temperature effects on the gonotrophic cycle of *Culicoides variipennis*. Journal of the American Mosquito Control Agency 7, 588–591.

[10] Paweska, J. T., Prinsloo, S., Venter, J., 2003. Oral susceptibility of South African *Culicoides* species to live-attenuated serotype-specific vaccine strains of African horse sickness virus (AHSV). Medical and Veterinary Entomology 17, 436–447.

[11] Roy, P., Bishop, D. H. L., Howard, S., Aitchison, H., Erasmus, B., 1996. Recombinant baculovirus-synthesized African horsesickness virus (AHSV) outer-capsid protein VP2 provides protection against virulent AHSV challenge. J. of Gen. Vir. 77, 2053–2057.

[12] Scanlen, M., Paweska, J. T., Verschoor, J. A., Van Dijk, A. A., 2002. The protective efficacy of a recombinant VP2-based African horsesickness subunit vaccine candidate is determined by adjuvant. Vaccine 20, 1079–1088.

[13] Scotter, D. R., Lamb, K. P., Hassan, E., 1971. An insect dispersal parameter. Ecology 52, 174–177.

[14] Venter, G. J., Paweska, J. T., 2007. Virus recovery rates for wild-type and live-attenuated vaccine strains of African horse sickness virus serotype 7 in orally infected South African *Culicoides* species. Medical and Veterinary Entomology 21, 377–383.

[15] Venter, G. J., Paweska, J. T., Van Dijk, A. A., Mellor, P. S., Tabachnick, W. J., 1998. Vector competence of *Culicoides bolitinos* and *C. imicola* for south african bluetongue virus serotypes 1, 3 and 4. Medical and Veterinary Entomology 12, 378–385.

[16] Wittmann, E. J., Mellor, P. S., Baylis, M., 2002. Effect of temperature on the transmission of orbiviruses by the biting midge, *Culicoides sonorensis*. Vaccine 20, 1079–1088.
